# Sustained use of biogas fuel and blood pressure among women in rural Nepal

**DOI:** 10.1016/j.envres.2014.10.031

**Published:** 2015-01

**Authors:** Maniraj Neupane, Buddha Basnyat, Rainald Fischer, Guenter Froeschl, Marcel Wolbers, Eva A Rehfuess

**Affiliations:** a Center for International Health, Ludwig-Maximilians-Universitaet, Munich, Germany; b Mountain Medicine Society of Nepal, Kathmandu, Nepal; c Oxford University Clinical Research Unit, Patan Hospital, Kathmandu, Nepal; d The Hospital for Tropical Disease, Wellcome Trust Major Overseas Programme, Oxford University Clinical Research Unit-Vietnam, Ho Chi Minh City, Vietnam; e Institute for Medical Informatics, Biometry and Epidemiology, Ludwig-Maximilians-Universitaet, Munich, Germany

**Keywords:** Biogas, Blood pressure, Cardiovascular disease, Household air pollution, Nepal

## Abstract

**Background:**

More than two fifths of the world's population cook with solid fuels and are exposed to household air pollution (HAP). As of now, no studies have assessed whether switching to alternative fuels like biogas could impact cardiovascular health among cooks previously exposed to solid fuel use.

**Methods:**

We conducted a propensity score matched cross-sectional study to explore if the sustained use of biogas fuel for at least ten years impacts blood pressure among adult female cooks of rural Nepal. We recruited one primary cook ≥30 years of age from each biogas (219 cooks) and firewood (300 cooks) using household and measured their systolic (SBP) and diastolic blood pressure (DBP). Household characteristics, kitchen ventilation and 24-h kitchen carbon monoxide were assessed. We matched cooks by age, body mass index and socio-economic status score using propensity scores and investigated the effect of biogas use through multivariate regression models in two age groups, 30–50 years and >50 years to account for any post-menopausal changes.

**Results:**

We found substantially reduced 24-h kitchen carbon monoxide levels among biogas-using households. After matching and adjustment for smoking, kitchen characteristics, ventilation status and additional fuel use, the use of biogas was associated with 9.8 mmHg lower SBP [95% confidence interval (CI), −20.4 to 0.8] and 6.5 mmHg lower DBP (95% CI, −12.2 to −0.8) compared to firewood users among women >50 years of age. In this age group, biogas use was also associated with 68% reduced odds [Odds ratio 0.32 (95% CI, 0.14 to 0.71)] of developing hypertension. These effects, however, were not identified in younger women aged 30–50 years.

**Conclusions:**

Sustained use of biogas for cooking may protect against cardiovascular disease by lowering the risk of high blood pressure, especially DBP, among older female cooks. These findings need to be confirmed in longitudinal or experimental studies.

## Highlights

• We study the impact of sustained use of biogas fuel on blood pressure among females.
• Use of biogas is associated with lower SBP and DBP in cooks >50 years.
• Use of biogas is associated with 68% reduced odds of developing high blood pressure in cooks >50 years.
• Effect of biogas use on blood pressure seems to be age dependent.

## 1 Introduction

More than two fifths of the world's population cooks with solid fuels, mostly in poor households of low- and middle-income countries (Bonjour et al., 2013). These fuels are often burnt in inefficient stoves inside poorly ventilated houses producing high levels of several health-damaging pollutants, in particular fine particles of a diameter of up to 2.5 µm (PM2.5) and mixture of other pollutants (Rehfuess, 2006, Naeher et al., 2007). In 2012, 4.3 million deaths were attributed to household air pollution (HAP) caused by such pollutants globally (WHO, 2014). Sixty percent of these deaths were due to cardiovascular complications-ischemic heart disease (IHD) and stroke (WHO, 2014), which are also the top two leading causes of global deaths (Lozano et al., 2012). The remaining 40% were due to adverse effects on respiratory health-mainly lower respiratory tract infections among children, and chronic obstructive pulmonary disease and lung cancer among adults (WHO, 2014). The overall disease burden due to HAP is much greater among women, as they tend to be primarily responsible for cooking and therefore receive higher exposures than other family members. Estimates using data from India show that reductions in HAP to WHO guideline limits would, for example, eliminate sixty percent more IHDs among women than among men (Smith et al., 2014).

Evidence linking HAP to cardiovascular disease (CVD) is recent, indeed there are no epidemiological studies that directly examine how HAP exposure increases the risk of IHD or stroke, although two studies have linked HAP exposure to the risk of high blood pressure (Mccracken et al., 2007, Baumgartner et al., 2011), which is a leading risk factor for global disease burden (Lim et al., 2012). Instead, the recent burden of disease estimates provided by the Global Burden of Disease 2010 project (Lim et al., 2012) and WHO (2014) are based on a novel integrated exposure-response analysis across multiple sources of particulate matter air pollution, ranging from active and passive smoking via HAP to ambient air pollution (Burnett et al., 2014). PM2.5 from ambient air pollution and active or passive smoking is a well-recognized risk factor for cardiovascular disease (Brook et al., 2010, Pope et al., 2009). The likely effect of HAP on CVD is inferential and based on similar physical characteristics of the particulates and HAP particulate matter exposures that are located in between active smoking and passive smoking/ ambient air pollution (Smith et al., 2014).

Likewise, no epidemiological studies have assessed whether switching to cleaner fuels could potentially translate into cardiovascular health gains in cooks previously exposed to high levels of HAP for a long period. A randomized trial in Guatemala produced evidence that reduced pollutant exposure after switching from an open fire to an improved stove could lower blood pressure in adult females (Mccracken et al., 2007) but this study could not measure the associated risk of hypertension due to its before-and-after intervention study design.

In rural Nepal, more than 85% of households are reliant on biomass fuels burnt using traditional stoves (CBS, 2012). Women in these households, who for cultural reasons start cooking from an early age, are exposed to very high levels of HAP. Peak concentrations of respirable particles inside kitchens at times are as high as 60,000 µg/m^3^ (Devakumar et al., 2014) while 24- h kitchen concentrations average several hundred µg/m^3^ (Kurmi et al., 2013, Singh et al., 2012).

There is limited evidence on the effectiveness of currently available improved stoves in reducing HAP exposure (Albalak et al., 2001, Riojas-Rodríguez et al., 2001). Therefore, a switch to clean fuels up to the next rung of the energy ladder appears to be the only way to meet WHO Air Quality Guidelines for PM10/PM2.5. Among rural Nepalese households, who depend on subsistence farming and animal rearing, the national Biogas Support Program (BSP) has been promoting an alternative source of household fuel for the last two decades. Biogas plants rely on anaerobic digestion of organic human and animal waste inside locally built underground digesters and produce gas rich in methane. This is easily piped to the kitchen and burnt for cooking and heating purposes. Around 300,000 such biogas plants have been adopted by rural households throughout the country (AEPC, 2013). However, to date neither the impact of this program on pollutants nor its impact on health have been examined in Nepal or globally.

We therefore conducted a propensity score matched cross-sectional study to explore if the adoption and sustained use of biogas plants by households impacts pollution levels and cardio-respiratory health compared to households that have continued to use traditional wood stoves. Specifically, we hypothesized that the sustained use of biogas for at least ten years would be associated with lower systolic and diastolic blood pressure and a reduced risk of hypertension among adult female cooks. The ten years lag time of biogas use was based on the hypothesis that the chronic effects of prior HAP exposure on the respiratory and cardiovascular system could take as long as a decade to normalize after switching to cleaner fuels.

## 2 Materials and methods

The protocol of this study was reviewed and approved by the Ethical Review Boards of the Nepal Health Research Council (Kathmandu, Nepal) and the Oxford Tropical Research Ethics Committee (University of Oxford, UK) prior to any contact with study participants. The Ludwig-Maximilians-Universitaet Ethical Commission (Munich, Germany) granted an ethical waiver for the study after having reviewed the two granted ethical clearances.

### 2.1 Study site

This study was carried out in Gorkha, a hilly district of Nepal, located 140 km west of the capital Kathmandu. To avoid the unwanted effect of traffic emissions, this district was purposively selected as it had the lowest road density network among the 19 districts with the highest rate of biogas adoption. Household recruitment was undertaken in four villages (Palungtar, Chyangli, Dhuwakot and Chhoprak), home to agricultural indigenous populations and located far away from industries, asphalted roads and mechanized traffic. All these villages were below 1000 m elevation from sea level and households used a combination of fuels: wood, biogas, liquefied petroleum gas (LPG), charcoal and small amounts of crop residues depending on season. Night time temperatures even in the winter never fell below freezing so families did not use space heating.

### 2.2 Sampling

Although we aimed for random sampling, the hilly terrain of the four villages, which were only accessible by foot, made it impractical to conduct a rapid census and determine a reliable sampling frame for biogas as well as wood users. Instead, we did a complete enumeration of all households who met the eligibility criteria and were within a radius of two hours of walking distance from the designated center of the villages.

### 2.3 Sample size

This study was primarily designed to evaluate the impact of sustained biogas use on the respiratory health of female cooks and was statistically powered to detect differences in FEV1 (forced expiratory volume in one second). As a secondary outcome we assessed cardiovascular health of the cooks and was powered to detect at least 5 mmHg differences in both the systolic as well as diastolic blood pressure between the two groups.

### 2.4 Study population and duration

All households of the selected villages with adult female cooks of 30 years or more with at least one cattle and having primarily cooked during the last ten years or longer with either biogas or traditional wood stoves were eligible. All recruitments were done during the summer months, 20 March–12 May, 2012 and 18 April–10 May, 2013.

### 2.5 Blood pressure measurement

In addition to the weight and standing height, we measured brachial blood pressure of participants at their homes. An automatic blood pressure monitor (Omron SEM-1; Omron Corp, Tokyo, Japan) was used to measure systolic and diastolic blood pressure. Participants were asked to rest in a chair or an improvised chair for 5 min with their feet flat on the ground and arms uncrossed at their side. The device automatically inflated and deflated and produced SBP, DBP and heart rate. Participants were kept at ease and were requested to remain quiet during the measurements. Measurements were repeated two minutes apart until three measurements within 10 mmHg were obtained. The average of three measures was used as the final blood pressure. Those with average SBP≥140 mmHg and/or DBP≥90 mmHg were considered hypertensive. Systolic hypertension was defined when SBP≥140 mmHg irrespective of DBP, and diastolic hypertension when DBP was≥90 mmHg irrespective of SBP.

### 2.6 Questionnaires

Four medical doctors and three students who had undergone specific training in community data collection techniques collected information on demographics of the cooks including their age, smoking status and education. Household characteristics like family size, number of children under the age of five years, number of rooms, number of cattle, type of material used for housing, access to drinking water and possession of durable assets like a toilet, radio, television and mobile phone were also documented. Kitchen characteristics including the type of stove, fuel use, eave spaces in kitchen walls, kitchen volume, number and dimensions of windows and doors were obtained by interview, observation and measurement. We also asked cooks about cardio-respiratory symptoms. Their body mass index (BMI) was calculated by dividing weight in kilograms by square of the height in meters.

## 3 Statistical analysis

We undertook double data entry in SPSS v 17 (SPSS Inc., Chicago) and any discrepancies were resolved referring to the original paper version of the questionnaire. Six participants with missing outcome information were excluded from the analysis. Crude between-group comparisons were based on the Wilcoxon test for continuous variables and Fisher's exact test for categorical ones.

We applied Principal Component Analysis (PCA) to generate a socio-economic score (SES) for each household based on ownership of household assets (radio, television and watch), number of animals (cattle, oxen, goats and pigs), housing materials (roof, wall and floor) and access to basic facilities (source of drinking water and toilet). Other variables (possession of mobile phone, land telephone, electricity, bicycle and scooter) with poor variability, i.e. when everybody or nobody owned a given asset, were excluded from the PCA. Such a composite score based on the first component obtained through PCA represents a stable, long-term measure of socio-economic status (Filmer and Pritchett, 2001) and is widely used, including in Demographic Health Surveys (Rutstein, 2008).

We stratified the cooks into two age groups, i.e. 30–50 years and more than 50 years, to account for the sudden increase in cardiovascular risk in post-menopausal women (Liu et al., 2001, Miller et al., 2007). A study among Chinese women had also identified age dependent effect of PM2.5 from HAP on blood pressure such that women 30–50 years and >50 years were differentially affected with a unit rise in exposure to log PM2.5 mass (Baumgartner et al., 2011). This age stratification is also supported by a large study which clearly showed the association between menopause and increased SBP and DBP independent of age and BMI (Zanchetti et al., 2005). In Nepal, studies have reported the mean age at which women attain menopause to be between 47 and 50 years (Rajbhandari et al., 2013, Marahatta, 2012, Aryal and Yadava, 2005).

Within each age group, we matched cooks using biogas to those using wood by their age, BMI and socio-economic score, with all of these variables used on a continuous scale. Age and BMI were chosen a priori to account for differential risk of high blood pressure, and SES to account for differential rates of biogas adoption. Full matching, a matching technique based on the concept of propensity scores, was used as it retains all the observations in the matched dataset (Hansen, 2004). This matching technique creates several fine subclasses of matched sets of cooks, allowing varying numbers of treated units (i.e. biogas users) and control units (i.e. wood users) in each (Ho D., Imai K., King G., Stuart E., 2006. MatchIt: Nonparametric Preprocessing for Parametric Casual Inference, R package version. vol. 2. pp. 2–11., Hansen, 2004). Balance was checked by assessing the reduction of absolute standardized bias (ASB) of matching covariates as well as other important ones. ASB is defined as the weighted difference in means of two groups divided by the standard deviation in the original treated groups. Balance was assumed to be well achieved when ASB was less than 0.25 (Ho et al., 2007, Stuart and Green, 2008). Jitter plots and histograms were additionally used to examine the distribution of propensity scores and weights assigned to each cook. All biogas-using cooks received a weight of one and firewood-using cooks received weights depending on the number of treated and control units in each matched subclass.

### 3.1 Analysis after matching

We used weighted linear and logistic regression models with the matching covariates included in the model to account for any remaining imbalances (Stuart and Green, 2008, Ho et al., 2007). SBP and DBP were the dependent variables in linear regression analyses. Similarly, hypertension, systolic hypertension and diastolic hypertension were the dependent variables in logistic regression models. In these regression models, we sequentially adjusted for (i) smoking status of the cooks (ever or never smoking), (ii) kitchen and ventilation characteristics (kitchen types, windows, eave spaces, kitchen volume) and (iii) additional fuel use. We used generalized estimating equation (GEE) methods in the regression models to take into account the correlation in outcomes of individuals, i.e. lack of independence within a matched subclass (Austin, 2008).

We performed sensitivity analysis by running the same regression models in unmatched datasets. Age, BMI and socio-economic score were included in the initial model; smoking and other variables were introduced sequentially as in the matched analysis.

All statistical analyses were performed in R version 3.0.2. (R Core Team, 2013). Matching was performed using the MatchIt package (Hansen, 2004, Ho D., Imai K., King G., Stuart E., 2006. MatchIt: Nonparametric Preprocessing for Parametric Casual Inference, R package version. vol. 2. pp. 2–11., Ho et al., 2007).

## 4 Results

We enrolled 519 cooks, one primary cook from each house, between 30 and 83 years of age. Table 1 shows the characteristics of cooks by fuel type and age group. Biogas users were comparatively older, had higher BMI and were of higher socio-economic status. Both age groups differed in the type and size of the kitchen they had. This is likely to be a consequence of the greater adoption of biogas plants by households with higher socio-economic status.

**Table 1:** Characteristics of cooks stratified by age group and primary fuel use.

Characteristics	30–50 years (*N*=297)			>50 years (*N*=222)		
	Wood (185)	Biogas (112)	*p* Value	Wood (115)	Biogas (107)	*p* Value
Age (years)	40 (35,45)	42 (38,47)	0.04	60 (55, 65.50)	61 (55, 67.50)	0.42
BMI (kg/m^2^)	21.93 (20.24, 23.96)	22.94 (20.74, 25.63)	0.02	20.48 (18.80, 22.52)	21.64 (19.27, 24.17)	0.03
Ever smokers	80 (43%)	29 (26%)	<0.01	88 (76%)	77 (72%)	0.45
Pack years smoked among smokers	6.58 (2.52, 11.81)	5.08 (2.48, 7.33)	0.33	10.00 (6.03, 20.00)	8.80 (4.43, 16.82)	0.31
Socio-economic status score (SES)	−0.51 (−1.56, 0.41)	0.97 (0.15, 1.89)	<0.01	−0.36 (−1.48, 0.42)	1.08 (0.41, 1.70)	<0.01
Kitchen volume (m^3^)	15.33 (8.97, 22.08)	21.20 (15.96, 26.60)	<0.01	17.35 (12.61, 2.35)	22.96 (17.75, 8.32)	<0.01
Kitchen type			<0.01			<0.01
Kitchen and living same	26 (14%)	13 (12%)		25 (22%)	10 (9%)	
Separate kitchen	78 (42%)	49 (44%)		45 (39%)	38 (36%)	
External kitchen	51 (28%)	49 (44%)		34 (29%)	55 (51%)	
Detached kitchen	25 (13%)	1 (1%)		9 (8%)	4 (4%)	
3 walled	5 (3%)	–		2 (2%)	–	
Eave spaces quality			0.07			0.12
Absent	104 (56%)	78 (70%)		70 (61%)	79 (74%)	
Poor	43 (23%)	20 (18%)		27 (23%)	18 (17%)	
Good	38 (21%)	14 (12%)		18 (16%)	10 (9%)	

Data are median (IQR) or number (%) unless otherwise specified. *p* Value based on Wilcoxon test for continuous data and Fisher’s exact test for categorical data (based on simulation with *B*=10,000 for large tables). All % are rounded and may not add up to 100%.

SBP progressively increased with age in this population as reported in other studies (Baumgartner et al., 2011). DBP increased up to the age of 50 years and then decreased thereafter. With both SBP and DBP measures, we observed a notch around the age of 50 years. The crude estimates of between-group comparisons of SBP, DBP and prevalence of hypertension are shown in Table 2. As expected, women aged more than 50 years had higher SBP and DBP and a higher rate of hypertension than women aged 30–50 years. In the older age group, one in two cooks had average blood pressure readings ≥140/90 mmHg, and the prevalence of high blood pressure in the overall population was 34.5%. Biogas users had lower overall SBP and DBP and also lower prevalence of hypertension than wood users; for DBP and diastolic hypertension, these differences were statistically significant. In the younger age group, biogas users had higher SBP and DBP than wood users but these differences were not statistically significant.

**Table 2:** Crude comparisons of blood pressure and prevalence of hypertension by age group and primary fuel use.

Characteristics	30–50 years (*N*=297)			>50 years (*N*=222)		
	Wood (185)	Biogas (112)	*p* Value	Wood (115)	Biogas (107)	*p* Value
SBP	121.33 (111.50,132.33)	124.00 (115.00,135.33)	0.07	139.00 (121.33,155.67)	136.50 (118.42,152.50)	0.44
DBP	78.67 (73.50, 86.33)	81.00 (74.50,87.00)	0.19	82.33 (76.33,92.00)	79.33 (73.42,87.67)	0.03
Hypertension	42 (23%)	25 (22%)	1	59 (52%)	51 (48%)	0.60
Systolic HTN	25 (14%)	20 (18%)	0.32	54 (48%)	48 (45%)	0.79
Diastolic HTN	33 (18%)	20 (18%)	1	36 (32%)	19 (18%)	0.02

Data are median (IQR) or number (%) unless otherwise specified. All % are rounded. *p* Value based on Wilcoxon test for continuous data and Fisher's exact test for categorical data (based on simulation with *B*=10,000 for large tables).

### 4.1 Impact of sustained biogas use on blood pressure among cooks older than 50 years

**Fig. 1:**
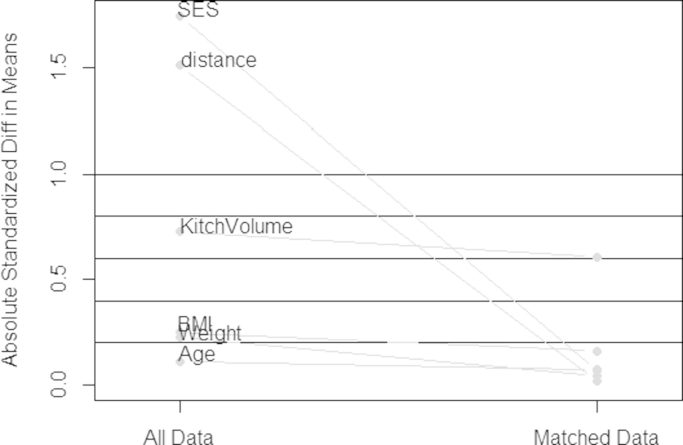
Balance achievement as shown by the reduction in absolute standardized bias after matching biogas users and wood users older than 50 years.

Fig. 1 shows the reduction in the absolute standardized bias and balance achieved for key variables after matching biogas-using cooks to wood-using cooks older than 50 years. The ASB for each of the matching covariates (age, BMI and SES) was reduced to less than 0.25. Before matching, participants differed significantly in their PCA-derived socio-economic scores. After full matching, participants were well balanced in their socio-economic score (ASB was reduced from 1.746 to 0.068) and distance measure (propensity score).

After matching and additional adjustments for smoking status, kitchen characteristics and additional fuel use, no statistically significant differences were observed for SBP. However, both the unadjusted and adjusted model yielded more than 6 mmHg lower average DBP among biogas users compared to wood users in this age group (Table 3).

**Table 3:** Unadjusted and adjusted differences in SBP and DBP in biogas vs wood-using cooks older than 50 years and matched by age, BMI and SES.

Matched by age, BMI and SES and additionally adjusted for	SBP (mmHg)		DBP (mmHg)	
	Difference (95% CI)	*p* Value	Difference (95% CI)	*p* Value
*Unadjusted effect of biogas*	−9.22 (−20.25, 1.81)	0.101	−6.14 (−11.56, -0.71)	0.027
Smoking	−9.45 (−21.06, 2.16)	0.110	−6.51 (−12.28, -0.74)	0.027
Smoking, kitchen characteristics, ventilation and additional fuel use	−9.84 (−20.43, 0.76)	0.069	−6.49 (−12.15, -0.82)	0.025

The adjusted and unadjusted estimates of the risk of developing hypertension are shown in Table 4. After matching and adjustment there was a 68% reduced odds (OR=0.32, 95% CI [0.14–0.71]) of developing hypertension among biogas users compared to wood users. Biogas users showed a substantial reduction (82%) in the odds of developing diastolic hypertension compared to their wood-using counterparts, whereas the observed differences in systolic hypertension were not statistically significant.

**Table 4:** Unadjusted and adjusted risks of developing hypertension in biogas vs wood-using cooks older than 50 years and matched by age, BMI and SES.

Matched by age, BMI and SES and additionally adjusted for	HTN		Systolic HTN		Diastolic HTN	
	Odds ratio (95% CI)	*p* Value	Odds ratio (95% CI)	*p* Value	Odds ratio (95% CI)	*p* Value
*Unadjusted effect of biogas*	0.40 (0.16, 1.00)	0.051	0.48 (0.16, 1.39)	0.178	0.25 (0.07, 0.88)	0.031
Smoking	0.39 (0.16, 0.92)	0.033	0.49 (0.17, 1.38)	0.176	0.23 (0.06, 0.83)	0.024
Smoking, kitchen characteristics, ventilation and additional fuel use	0.32 (0.14, 0.71)	0.005	0.42 (0.17, 1.02)	0.054	0.18 (0.05, 0.60)	0.005

### 4.2 Impact of sustained biogas use on blood pressure among cooks aged 30– 50 years

**Fig. 2:**
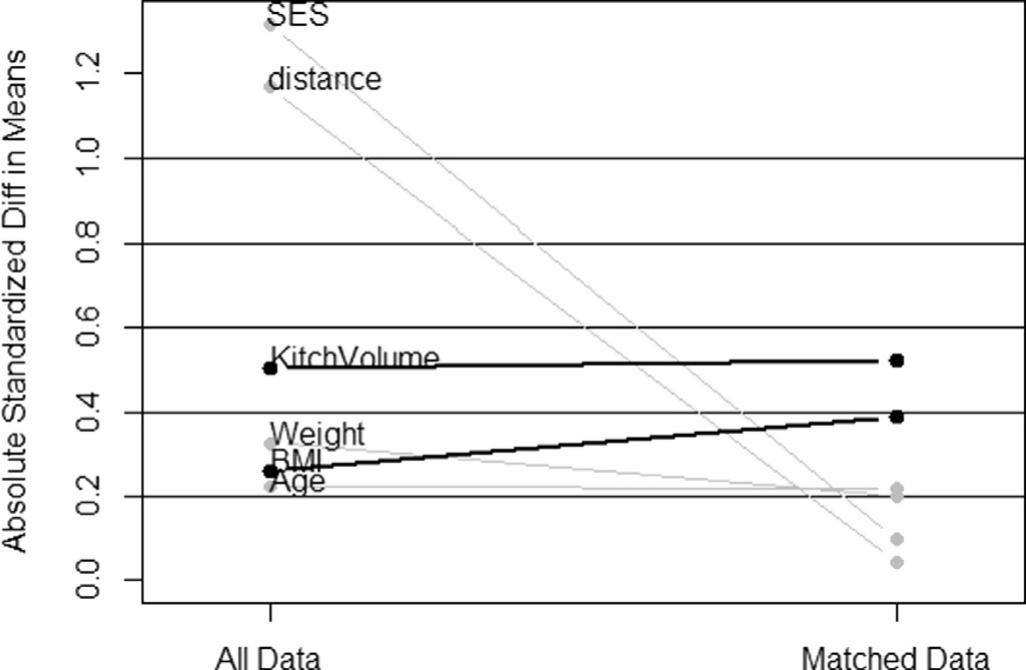
Balance achievement as shown by the reduction in absolute standardized bias after matching biogas users and wood users aged 30–50 years.

In the younger age group, however, balance was not well achieved using the same a priori agreed set of matching covariates (Fig. 2). The ASB for BMI actually worsened from 0.262 in the unmatched dataset to 0.387 after matching and age displayed only minimal improvement (from 0.223 to 0.217). SES and the overall distance measure (propensity score) were well balanced, with the ASB for SES being reduced from 1.319 in the unmatched dataset to 0.099 in the matched dataset.

Contrary to older cooks, the adjusted matched analysis in the younger group showed a statistically significant 4 mmHg higher SBP among biogas users compared to wood users but no differences for DPB (Table 5).

**Table 5:** Unadjusted and adjusted differences in SBP and DBP in biogas vs wood-using cooks aged 30–50 years and matched by age, BMI and SES.

Matched by age, BMI and SES and additionally adjusted for	SBP (mmHg)		DBP (mmHg)	
	Difference (95% CI)	*p* Value	Difference (95% CI)	*p* Value
*Unadjusted effect of biogas*	3.59 (−0.77, 7.95)	0.106	1.14 (−2.04, 4.32)	0.482
Smoking	3.76 (−0.37, 7.89)	0.075	1.37 (−1.64, 4.39)	0.372
Smoking, kitchen characteristics, ventilation and additional fuel use	4.38 (0.86, 7.90)	0.015	1.49 (−0.95, 3.93)	0.231

Table 6 shows the risk of hypertension among younger cooks, where no statistically significant differences between the two groups of fuel users were observed.

**Table 6:** Unadjusted and adjusted risks of developing hypertension in biogas vs wood-using cooks aged 30–50 years and matched by age, BMI and SES.

Matched by age, BMI and SES and additionally adjusted for	HTN		Systolic HTN		Diastolic HTN	
	Odds ratio (95% CI)	*p* Value	Odds ratio (95% CI)	*p* Value	Odds ratio (95% CI)	*p* Value
*Unadjusted effect of biogas*	0.96 (0.37–2.44)	0.925	0.94 (0.32–2.70)	0.901	1.14 (0.38–3.39)	0.811
Smoking	1.08 (0.46–2.52)	0.857	1.07 (4.16–2.74)	0.890	1.32 (0.51–3.41)	0.572
Smoking, kitchen characteristics, ventilation and additional fuel use	1.66 (0.75–3.72)	0.213	1.67 (0.75–3.72)	0.213	1.78 (0.67–4.76)	0.247

An unmatched sensitivity analysis yielded results that were similar to those of the matched analysis. However, the effect sizes were lower in both age groups and did not always reach statistical significance.

## 5 Discussion

We conducted an epidemiological study to assess the real-life impact of biogas interventions on the risk of hypertension in rural Nepal. Importantly, this reflects impact on blood pressure and likely cardiovascular disease risk that is achievable when households self-select to adopt and use biogas plants to meet their cooking needs under the Nepalese Biogas Support Program.

To account for the non-random distribution of self-adopted interventions, we used both propensity score matching (PSM) and statistical regression model adjustment to account for potential confounding. PSM is widely used in the analysis of observational studies but rarely used in HAP studies. It aims to yield unbiased effect estimates of non-randomly distributed interventions by balancing the covariates distribution between the treated and the untreated groups such that treatment assignment (i.e. adoption and sustained use of biogas in our context) is ignorable (Rosenbaum and Rubin, 1983, Williamson et al., 2012). In our case, the matched analysis yielded differences associated with fuel type with less bias owing to reduced ASB of the matching covariates, age and BMI, which are both biological determinants of blood pressure. Matching by SES helped to overcome selection bias in self-adoption of biogas plants. Before matching, participants differed significantly in their PCA-derived SES scores. After full matching, participants were well balanced in their SES.

Applying the concept of PSM in the impact assessment of self-adopted biogas interventions, we found sustained use of biogas for at least ten years associated with lower levels of SBP and DBP among cooks beyond the fifth decade of their life. We also observed significantly reduced odds of developing hypertension among biogas users>50 years. This was, however, not observed in the younger age group. Although we do not discount the possibility that biogas may not have any effect (or only a very small effect) in younger subjects, this could rather be due to persistent large imbalances in matching covariates, in particular in relation to BMI, even after matching. BMI imparts a significant effect on blood pressure (Kaufman et al., 1997), and the worsened balance of this covariate may lead to an estimate of the effect of biogas use which is biased towards the null.

The lack of comparable studies assessing the impact of biogas use on blood pressure prevents us from comparing our findings. However, our results are consistent with those of the randomized trial in Guatemala which observed reduction in SBP and DBP after switching from an open fire to a *plancha*-improved woodstove with chimney. We observed 9.8 mmHg (95% CI, −20.4 to 0.8) and 6.5 mmHg (−12.15 to −0.82) lower SBP and DBP in biogas users while the differences observed in Guatemala were 3.7(−8.1 to 0.6) mmHg and 3.0(−5.7 to −0.4) mmHg respectively. The higher average reduction, although overlapping confidence interval, in blood pressure in our study could be explained by decade-long use of biogas fuel for cooking as opposed to participants in the Guatemala study who had used improved *plancha* stoves for less than a year (293 days, 2–700 days) (Mccracken et al., 2007). We also observed substantially lower 24 -h kitchen concentrations of carbon monoxide (while the PM2.5 data is still being analyzed owing to the lack of adjustment factor for biogas fuel) among the households using biogas (manuscript in preparation), with the observed reductions similar to those achieved with *plancha* stoves.

A study from China similar to ours also observed age-related effects of HAP on blood pressure. They reported a 4.1 mmHg and 1.8 mmHg increases in SBP and DBP respectively associated with each one log unit increase in PM2.5 mass among >50 years old cooks; as in our study, the Chinese study failed to identify similar associations among cooks aged ≤50 years (Baumgartner et al., 2011). For these younger cooks, the Chinese study reported a lowering of DBP with increased PM2.5, although not significant, and a very small effect on SBP while both SBP and DBP tend to be higher among young biogas users in our case.

We found a very high prevalence (35%, 177/513) of high blood pressure in this population similar to the prevalence reported by Sharma et al. (2011) in a large community-based screening program (34%). Other studies conducted in Nepal also reported similar prevalences of hypertension of 31% (Ministry of Health and Population, 2008) and 34% (Vaidya et al., 2012). However, HAP studies in neighboring China and India reported only 13% (Baumgartner et al., 2011) and 20% prevalence (Dutta et al., 2011) respectively. It is noteworthy that both studies showed very different characteristics, with the Indian study studying only pre-menopausal women and the Chinese study including only non-smokers. These important differences in population characteristics are likely to be the main reason for the stark contrast in prevalence observed in these studies compared to ours.

High prevalence of smoking among the cooks is of potential concern as it may dilute the effect estimates for biogas. However, both fuel groups had similar rates of “ever smokers” and median number of pack-years of cigarettes smoked (7.5 years, *p*=0.61). Additionally, exposure through neighborhood air pollution could also contribute to a reduction in the effects observed for biogas and therefore bias the effect estimate towards the null.

Exposure to PM2.5 has been found to cause high blood pressure through interplay of intermediary pathways – mainly vascular inflammation and oxidative stress, autonomic nervous imbalance, endogenous release of mediators or direct vascular action of PM (Brook and Rajagopalan, 2009). High blood pressure is a major risk factor for atherosclerosis at all ages (Fausto et al., 2004). Besides, exposure to PM has been found to increase tendency for coagulation and platelet activation thus further favoring atherosclerosis (Brook et al., 2004). More than 90% patients with IHD have atherosclerosis of one or more of the coronary arteries (Fausto et al., 2004). Detailed pathway of how HAP, in particular the PM2.5 component of HAP, is potentially linked to CVD risk is discussed in detail elsewhere (Brook et al., 2004, Brook et al., 2010, Pope and Dockery, 2006). But it is important to underscore that the exposure-response for CVD is non-linear and the risk increases steeply at lower exposures and flattens out at higher exposures (Pope et al., 2009). This has important programmatic implications such that clean interventions should aim to get HAP exposure to a very low level to bring about meaningful reductions in the risk of CVD.

Our findings are of public health importance given that, globally, 2.8 billion people are exposed to HAP (Bonjour et al., 2013) and more than a billion people have high blood pressure (WHO, 2011), thereby increasing their CVD risk substantially. Besides, if the magnitude of our effect estimates are confirmed, this has large health implications given that a 10 mmHg lower SBP or 5 mmHg lower DBP is associated with an approximately 25% lower risk of coronary heart disease and 40% lower risk of stroke (Asia Pacific Cohort Studies Collaboration, 2003, Macmahon et al., 1990). The BSP program in Nepal which still has a large potential to scale up biogas adoption throughout the country could bring forward health perspective in the promotion of biogas by citing the potential health advantages associated with sustained biogas use. In India and China, more than 46 million household biogas plants were already installed by 2011 (GACC, 2012) and these results could further give an impetus to drive the program in even a larger scale.

### 5.1 Strengths and limitations

Because of the cross-sectional design of this study, estimates of the effect of biogas intervention on the risk of hypertension should not be assumed to be causal. However, unlike controlled efficacy studies, this is a pragmatic study assessing the impact of self-adopted interventions in the community. Hence our findings may be more appropriate in reflecting of the true field performance of an intervention adopted and used by the households in the community without undue external “push”.

We employed a purposive sampling in selecting study villages. So, generalizability of our findings may be limited to similar rural villages. However, these remote rural villages were selected to avoid undue contamination of the effect of HAP reductions through major exposures to industrial emissions as well as vehicular exhaust fumes. At the village level, all households within a defined distance radius were recruited. Owing to the difficulty of determining a sampling frame in remote hilly villages, a full enumeration of all village households and random sampling were not feasible thus households from the very remote parts of the village were missed as they were unreachable with time constraints and logistic issues. These parts are more remote and are therefore less likely to adopt biogas plants being further away from availability and supply of biogas parts and promotional information about biogas.

Biogas users in our study adopted their plants by themselves and might have been influenced by the knowledge of possible health benefits with reduced smoke and lead to biased effect. If so, biogas users – apart from having a higher socio-economic status – may be more health-aware and healthier than non-biogas users, with this health awareness influencing their cardiovascular disease profile in positive ways and potentially exaggerating the true effect of biogas use. However, a significant proportion of households in this study adopted these plants motivated by a lack of firewood rather than any perceived health advantages. Awareness of the health impact of household air pollution was stated by less than 5% of biogas users as one of the reasons for fuel switching (manuscript in preparation).

The two population differed substantially with respect to observed characteristics including SES, kitchen volume etc. and might also differ with respect to unmeasured characteristics. While we aimed to use matching and regression adjustment to account for these differences, there still remains a possibility of bias due to unmeasured confounding. We did not collect details of daily salt intake, physical activity, secondhand smoke exposure and anti-hypertensive medication use. We also did not inquire about the family history of hypertension owing to the difficulty in collecting this history precisely from our rural study population, where 77% of the participants were illiterate and this remained uncontrolled in our analysis. Additional risk factors like excessive alcohol intake and stress were also not measured. If these factors were to be differentially distributed between the two fuel groups, presumably with healthier lifestyles associated with higher SES among biogas users, our results would be biased away from the null. We believe, however, that these behaviors and additional risk factors were relatively homogenously distributed among biogas vs wood users in these remote rural villages given the socio-economic structure of the agricultural indigenous population.

Although we report substantially reduced CO inside the kitchens with biogas use, PM2.5 may be the prime exposure of interest related to our outcome. Availability of PM2.5 data would have further strengthened our study but it is likely that the use of gaseous fuel biogas also achieves reduction in PM2.5 along with CO.

Although blood pressure was measured using standardized protocols, participants were not re-evaluated on another occasion to confirm their hypertension status. This might have led to a residual misclassification of the outcome; however this misclassification should be non-differential in both groups. Similarly, the un-blinded exposure status of the cooks is unlikely to influence our findings as we used automatic blood pressure machines to measure blood pressure. In terms of addressing the potential confounders of BP, we have addressed menopause-related effects on BP by age stratification and avoided any issue of seasonal variation in BP by recruiting all participants during the summer months. Additionally, all participants lived in a household together with their family members thus ruling out any effect of living alone on BP.

Findings from our study give evidence that the Nepal Biogas Support Program has been successful in reducing the cardiovascular health risks among its female users. In the context where 80% of the Nepalese population resides in rural areas with similar socio-cultural practices and beliefs, and relying mainly on subsistence farming and animal rearing, findings from this study conducted in four rural villages are likely to be observed in other similar rural settings as well. These results are also of broad importance to other biogas programs mainly of neighboring China and India where more than 46 million household have already adopted such plants. Differences in cooking preferences and cooking needs in these countries may result in findings contrary to ours so well-designed longitudinal studies in different socio-cultural practices should be carried out before a generalizabilty can be assumed to broader population.

## 6 Conclusion

Sustained use of biogas for cooking for at least ten years is associated with lower systolic and diastolic blood pressure as well as reduced risk of developing hypertension in female cooks older than 50 years. Although the findings of our study need to be confirmed by well-designed longitudinal studies, our study suggests that household biogas plants could be an alternative energy source to improve cardiovascular health of millions of cooks exposed to household air pollution in other parts of Nepal and elsewhere in the world.

## Funding

This study received joint funding from the Center for International Health, Munich which funded the equipment and the Wellcome Trust of Great Britain which funded the field work section of the study and supported the statistical analysis. The sponsors of the study had no role in study design, data collection, data analysis, data interpretation, or submission of manuscript.

## Author’s contribution

ER, MN, RF and BB conceived and designed the study, MN collected field data with supervision from ER, BB, RF and GF. MN analyzed the data with help from MW and ER. MN wrote the first draft of the paper and all authors contributed in the revision. The final manuscript was read and approved by all the authors.

## Conflict of interest

None.
